# Supplementary material for: Chromothripsis during telomere crisis is independent of NHEJ, and consistent with a replicative origin
Source: Genome Res. 2019 May;29(5):737–49. doi: 10.1101/gr.240705.118 (PMC6499312; doi:10.1101/gr.240705.118)
Supplement: Supplemental Material [file supp_gr.240705.118_Supplemental_file_1.zip › contigs/annotated_contigs/DB82/contig.4.DB82_length_512_mean_cov_14.017578125.docx]

**DB82_length_512_mean_cov_14.017578125**

```
ATATAATCTAGGATTTTAGATCTAGCAATTTTGTGTGTGTGTGTGTGTTTCTTCTCTTTTAAAAATATTCATGATATTTATTATATTTA
 >chr7:31669475-31669647 - E=7e-86
TATCATGTTTGTATATTTTACAATTCTTTAGACTTTGCCTTACCTGTCCTTTTAAGCAAGACTTCCTAATTCAGTACGCTGGTGTAC|T

TGTTATA|TGAGATAACAATTTATGATGCAGTTCAAAATCAGGCCTACTTAAATGTGAAGTTATACTCTGTCTT|TATT|TACCAATCA
        >chr7:31669975-31670045 + E=2e-30                                 >chr7:316702
GTTATAAAAATAGATATAAAATTACAAACAATAAGACTAAATATATAGCTTATGAAAATA|TA|GTTCAGAGGGCACAACCTAATGATA
38-31670311 + E=3e-32                                          >chr7:31668929-31669116
ACTGTTTCTATTCCCCCTCCCTACAAGCTGTGTTTCTGTCCAGGGGAAGGAAAACAGGCCATTGTGCCTGGAGCTTCATGGCATGGAGT
- E=7e-98
AATCAGGGGTTTGGTAATGCAGGGACTTGGAATGAAGCAAGTTAGAATCTTAATCCAATTAAATATTTCTGAA
```
